# Immunostimulation by Synthetic Lipopeptide-Based Vaccine Candidates: Structure-Activity Relationships

**DOI:** 10.3389/fimmu.2013.00318

**Published:** 2013-10-09

**Authors:** Mehfuz Zaman, Istvan Toth

**Affiliations:** ^1^School of Chemistry and Molecular Biosciences, The University of Queensland, St Lucia, QLD, Australia; ^2^School of Pharmacy, The University of Queensland, St Lucia, QLD, Australia

**Keywords:** self-adjuvanting, lipopeptide, vaccine, peptide, toll-like receptor

## Abstract

Peptide-based vaccines offer several advantages over conventional whole organism or protein approaches by offering improved purity and specificity in inducing immune response. However, peptides alone are generally non-immunogenic. Concerns remain about the toxicity of adjuvants which are critical for immunogenicity of synthetic peptides. The use of lipopeptides in peptide vaccines is currently under intensive investigation because potent immune responses can be generated without the use of adjuvant (thus are self-adjuvanting). Several lipopeptides derived from microbial origin, and their synthetic versions or simpler fatty acid moieties impart this self-adjuvanting activity by signaling via Toll-like receptor 2 (TLR2). Engagement of this innate immune receptor on antigen-presenting cell leads to the initiation and development of potent immune responses. Therefore optimization of lipopeptides to enhance TLR2-mediated activation is a promising strategy for vaccine development. Considerable structure-activity relationships that determine TLR2 binding and consequent stimulation of innate immune responses have been investigated for a range of lipopeptides. In this mini review we address the development of lipopeptide vaccines, mechanism of TLR2 recognition, and immune activation. An overview is provided of the best studied lipopeptide vaccine systems.

## Introduction

The immune system functions to defend against invasion by pathogens. Vaccination is predominantly a preventive measure that aims to build specific immune defenses (antibodies or cellular) to protect an individual against infection. Traditional vaccine formulations are based on whole (inactivated or killed) pathogens or pathogen-specific proteins. While this model of vaccine has been successful for preventing some infectious diseases, many pathogens cannot be targeted using this approach. Problems include undesirable host reactions, reversion to virulence, and the inability to efficiently culture the pathogen. Logistical problems faced by traditional vaccines include the necessity to maintain a cold chain: an uninterrupted series of refrigerated storage, transport, and distribution (1).

**Figure 1:**
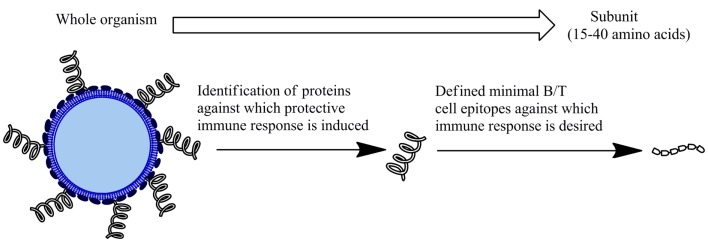
**Subunit vaccines**. An alternative to inactivated or attenuated whole pathogen vaccines. Comprised of fragment/s of the microorganism to generate a protective immune response.

Subunit vaccines based on short, specific fragments of a pathogen provide a rational and attractive alternative to traditional vaccine design (Figure 1). Rational vaccine design are composed of antigen(s), delivery systems, and adjuvant that induce immune responses against specific epitopes to protect against a pathogen (2). These vaccines are safer as they are non-infectious and more immunologically defined than traditional approaches. Synthetic peptides have received considerable interest as a basis for subunit vaccine design. Peptide subunit vaccines are generally composed of 30–60 amino acids representing the antigen/s of interest. Peptide/s that represent the minimal component of a protein against which an immune response is desired enables more precise selection of vaccine components, allowing for specificity of immune responses. Peptides are directly involved in the extent and specificity of cellular (T cells) and humoral (antibody) immune responses as this is generally mediated through the recognition of peptides representing B cell receptor (BCR) and T cell receptor (TCR) epitopes (2). Therefore, delivering pathogen-derived peptides that are B and/or T cell receptor epitopes represents an effective way induce an immune response. Furthermore, peptides can easily be synthesized and characterized and are generally more stable than whole pathogens or proteins. Studies have shown peptide-based vaccine candidates to be effective following ambient temperature storage (3). This lowers the need to maintain a cold chain, which is of significance particularly in developing countries that lack expensive refrigeration capability. This combination of generating an effective, specific immunity, and providing an economical alternative to current vaccination programs makes subunit peptide-based vaccine development highly promising.

Although representing promising vaccine candidates, peptides are often poorly immunogenic when used alone as vaccines. This is due to the lack of pathogen-derived molecules to act as danger signals that are required for efficient activation of the innate immune system, which consequently leads to lack of an adaptive immune response. Peptide-based vaccines require additional adjuvants to be effective. Adjuvants are defined as any agent that can increase the immunostimulatory effect of an antigen. In this review, after briefly introducing the key immunological interaction between vaccines and the immune system, the latest developments in the design of lipopeptide-based vaccines will be discussed.

## Main Immunological Concepts of Vaccine Development

**Figure 2:**
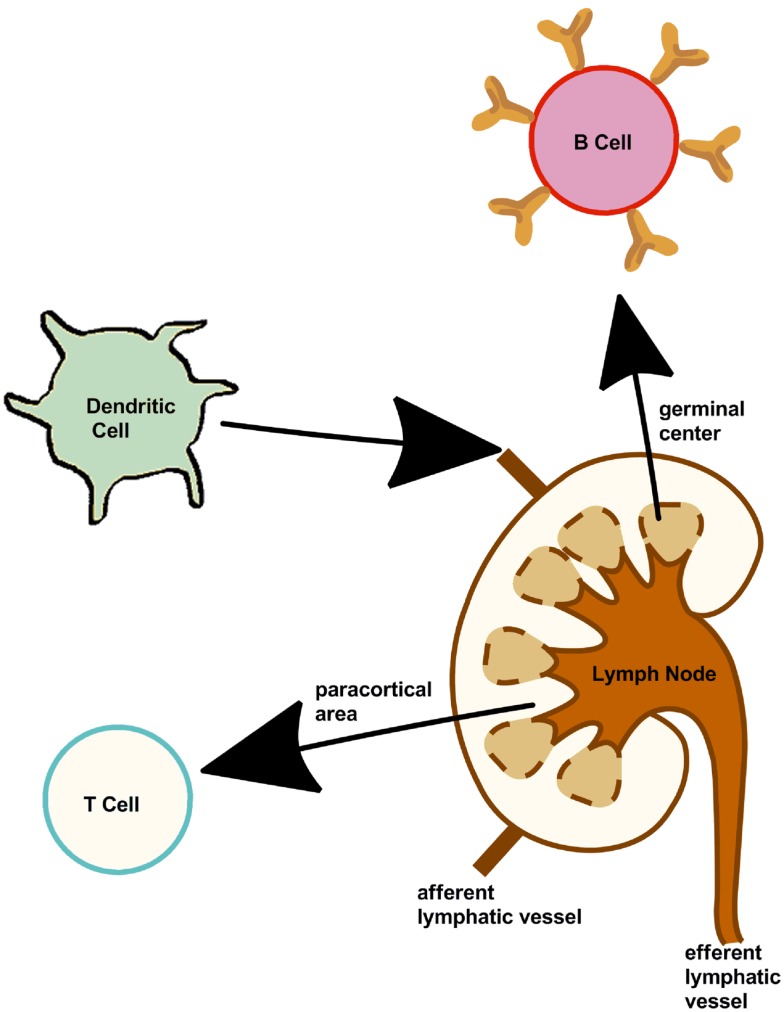
**Cells of the immune system**. Lymph nodes present along the lymphatic vessels contain specialized compartments where immune cells collect and encounter antigens. B, T, dendritic cells, and other immune cells are present, thus can mount an effective and appropriate immune response.

Adaptive immunity is based on two components, one responsible for the cytotoxic immune response (cellular) the other for generating the humoral (antibody) immune response. The three main cells [B cells, T cells, and dendritic cells (DCs)] involved in inducing an immune response are summarized in Figure 2. The is “adaptive” immune response is named as such as it provides the host with the ability to identify and memorize specific pathogens (to generate immunity) and to mount a more effective response when the pathogen is re-encountered. This mechanism allows a small number of genes to generate a vast number of different antigen receptors and the propagation of memory B cells and T cells that are the essential to long-term pathogen-specific immunity. This is also known as immunological memory. It is the memory cells form a pool of effective B and T cells and upon interaction with a previously encountered antigen, the appropriate memory cells are selected and activated.

Cytotoxic T lymphocytes (CTLs; also called CD8+ T cells) exert cytotoxic action on infected or tumor cells. Humoral immune responses are based on B cells which generate antibodies against pathogen-specific components. Both cellular and humoral immune responses require T-helper cells (TH cells; also called CD4+ T cells) (4). TH cells have an important role in adaptive immunity: through release of cytokines they mediate B cell antibody class switching and activation and proliferation of CTLs (5, 6). TH cells have been further subdivided into TH1 or TH2 lineage according to their cytokine profiles. TH1 cells are associated with enhancing clearance of intracellular pathogens and are defined on the basis of their production of the cytokine interferon-gamma (IFN-γ) (7). TH2 cells are essential for the control of certain parasitic infections and mediate host defense against extracellular parasites through the production of interleukin 4 (IL-4), IL-5, and IL-13.

**Figure 3:**
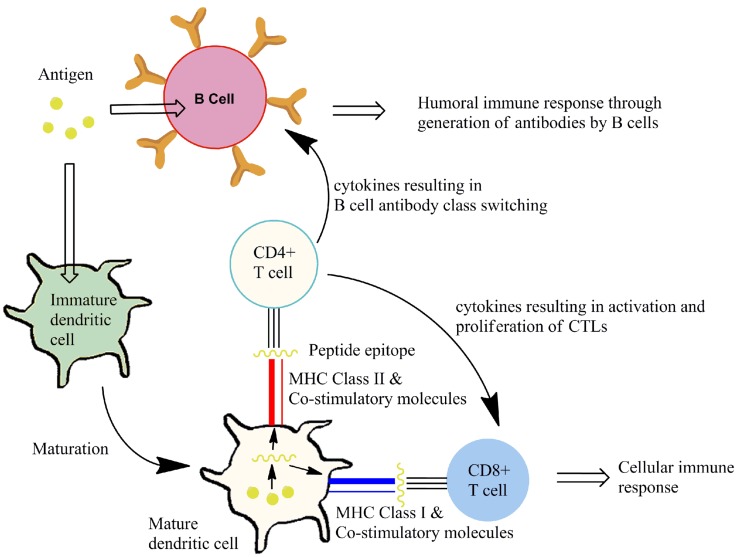
**Induction of an immune response**. Antigen is taken up by antigen-presenting cells (e.g., DCs), in the presence of immunostimulatory molecules, resulting in DC maturation. This maturation process involves processing of antigen to display peptide epitopes on MHC class molecules alongside co-stimulatory molecules to T cells. Subtypes of T cells that recognize the peptide-epitope then mediate an antigen-specific cellular or humoral immune response.

Cytotoxic T lymphocytes recognize infected or cancerous cells through recognition peptides from within the cell. These peptides are displayed on major histocompatibility complex class I (MHC class I) molecules. Presentation of appropriate peptide epitopes on MHC class I molecules by specialized antigen-presenting cells (APCs), such as DCs and macrophages, results in a cellular response. TH cells recognize peptides derived from extracellular proteins displayed on MHC class II molecules on APCs. Recognition by receptors on TH cells the MHC class II bound peptides allows interaction with the APC. Additional interactions occur through co-stimulatory molecules that are expressed on APCs and their ligands on the TH-cell. These events, known as activation signals, allow the TH-cell to respond to B cells that have taken up and display the same peptide/MHC II complexes. It is this interaction between TH cells and B cells that result in differentiation of B cells into plasma cells that secrete antigen-specific antibodies. Some cytokine signals sent by TH cells stimulate CTLs. In both cellular and humoral responses, the TCR on the surface CTL and TH-cell associates with the MHC I/peptide-epitope or the MHC II/peptide-epitope respectively. This peptide dependent recognition processes results in propagation of immune responses that control extracellular/intracellular infection. This is represented in Figure 3. The goal of vaccination is to induce protective immunity by stimulating antigen-specific CTLs or B cells with the help of TH cells. Therefore, vaccine components must contain two antigenic epitopes: a TH epitope and an antigen to elicit an antigen-specific B cell or CTL response, as desired.

## Adjuvants for Peptide-Based Vaccines

In addition to the selected B or CTL epitopes and TH-cell epitopes, peptide-based vaccines require an adjuvant to be immunogenic. Adjuvants can activate the immune system in a variety of ways: protecting antigen from rapid degradation; providing an antigen depot which allows antigen sampling by APCs for a longer time before clearance; by activating pattern recognition receptors (PRRs) on APCs which recognize pathogen-associated molecular patterns (PAMPs). Although numerous vaccine adjuvants have been reported, few have been approved for human use. Adjuvants licensed for human use include alum, monophosphoryl lipid A (MPL), virus-like particles, and immunopotentiating reconstituted influenza virosomes (IRIV) (1). In Europe, MF59 and AS03 (oil-in-water emulsions) are licensed for use for influenza vaccines (1). AS04, a combination of MPL and alum, is approved for hepatitis B virus (HBV) and human papillomavirus (HPV) vaccines in Europe and the USA (1). Adjuvants licensed for human use are listed in Table 1. Many of the aforementioned adjuvants approved for human use are unsuitable for peptide vaccines. Recent advances in development of adjuvants, including agonists for PRRs have made peptide-based vaccines a promising option for development of modern vaccines.

**Table 1:** **Adjuvants licensed for human use including the year licensed and the propieratory company, composition and use in relevant vaccines (8)**.

Name	Composition	Vaccine
Alum (1924)	Aluminum phosphate or aluminum hydroxide	Numerous
MF59 (Novartis, 1997)	Squalene, polysorbate 80, sorbitan trioleate	Seasonal, pandemic, and pre-pandemic influenza
AS03 (GlaxoSmithKline, 2009)	Squalene, tween 80, α-tocopherol	Pandemic and pre-pandemic influenza
Virosomes (Berna Biotech, 2000)	Lipids, hemagglutinin	Seasonal influenza, hepatitis A
AS04 (GlaxoSmithKline, 2005)	Aluminum hydroxide, MPL	Hepatitis B, human papilloma virus

## Toll-Like Receptors

**Figure 4:**
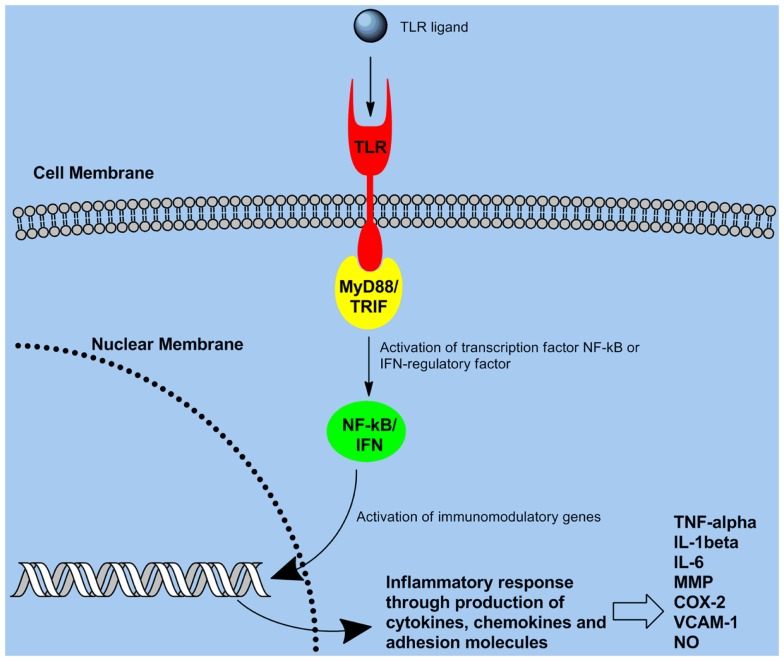
**Toll-like receptor signaling pathway: recognition of pathogens through TLR ultimately triggers signaling through the NF-κB pathway, consequently leading to gene activation and production of pro-inflammatory cytokines/chemokines**.

Toll-like receptors (TLRs) are the most thoroughly characterized PRR which are expressed on variety of cells including B cells (9), CD4+ T cell subsets (10), monocytes (11), APCs (DCs, and macrophages) (12), and certain types of epithelial cells (13). TLR expression patterns on these cells can vary. Most TLR ligands are microbial products (representing PAMPs) that signal the presence of an infection to the host. TLR-mediated stimulation of APCs significantly enhances the secretion of pro-inflammatory cytokines, antibody production, and immune responses. TLRs are evolutionarily conserved proteins, characterized by an extracellular leucine-rich repeat domain and an intracellular Toll/IL-1 receptor-like (TIR) domain (1). TLRs utilize activation of intracellular adaptor molecules and kinases to induce an inflammatory response (1). The transcription factor NF-κB is a key regulator of the expression of pro-inflammatory mediators that leads to an immune response (2). Numerous genes with roles in immunity and inflammation are regulated by NF-κB. Associating the TIR domain of the adaptor molecule MyD88 with the TIR domain of TLRs is needed for signaling to NF-κB/mitogen-activated protein kinase pathways Figure 4 (6, 14). This consequently results in MyD88 engaging IL-1 receptor-associated kinase, which then induces activation of tumor necrosis factor receptor-associated factor 6, NF-κB, and mitogen-activated protein kinases (6). Although MyD88 is a universal adaptor protein for all TLRs (5), studies revealed the existence of four other adaptor proteins named MAL, TRIF, TRAM, and SARM which can be used in a TLR-specific way (3, 6, 15, 16). A basic overview of the TLR signaling pathway is provided in Figure 4.

Ten human and 12 murine TLRs have been identified, TLR1-10 in humans, and TLR1**-**9 and TLR11**-**13 in mice (17). Mouse TLR10 is a pseudogene because only a partial (non-functional) genomic sequence has been detected and it is not expressed (18). TLR PAMPs include: bacterial cell-wall components, e.g., lipopolysaccharide (LPS) (19), peptidoglycan (20), and lipopeptides (21); flagellin (22); bacterial DNA (23); and viral RNA (24). Many of the TLR ligands were originally identified through screening of PAMPs in human embryonal kidney HEK293 cells transiently transfected with TLRs (25–27). HEK293 T cells present a valuable model for these studies as they do not naturally express any TLRs (25, 28). From 1999 onward, Akira and colleagues have generated multiple TLR and adaptor molecule-knockout mice (29). This has been invaluable for defining *in vivo* specific ligands for each TLR and defining the important roles of TLRs in immunity.

The use of synthetic peptides for vaccine development has been hampered by problems such as need for an adjuvant. The incorporation of lipopeptides as a strategy to enhance immunogenicity has been ongoing since the early 1980s. The following sections expand on the development of lipopeptides as vaccines and their TLR2-targeting ability as the basis for their immunogenicity.

## TLR2 and Lipopeptides

A membrane surface receptor, TLR2 recognizes many bacterial, fungal, and viral molecules. Generally this result in the uptake of TLR2 bound molecules and cellular activation of APCs (30–32). TLR2 recognizes lipoteichoic acid (33–35), zymosan (36, 37), and bacterial lipoproteins (38–40). TLR2 has also been reported to recognized peptidoglycan (41, 42), however this sensing was lost after the removal of lipoproteins or lipoteichoic acids (43).

**Figure 5:**
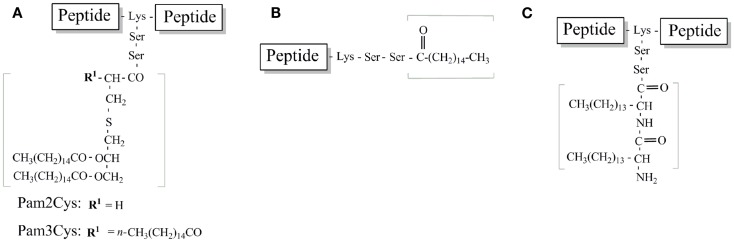
**Schematic structure of TLR2-targeting lipopeptides used in peptide vaccine development**. **(A)** Pam2Cys and Pam3Cys lipidated peptide. **(B)** Palmitoylated peptide **(C)**. Lipoamino acid-based peptide.

The selection of TLR2-targeting adjuvants for peptide vaccines has focused on bacterial lipopeptides and their synthetic analogs (Figure 5). These are common bacterial cell-wall components. A number of bacterial species produce lipopeptides that have important biological functions (44). They generally consist of short structures of amino acids linked to fatty acids via ester or amide bonds. Gram-positive bacterial lipoproteins contain two fatty acid chains compared to three in Gram-negative lipoproteins. The acyl chains are heterogeneous in terms of their length and degree of saturation, however most frequently they are palmitic acid (containing 16 carbons). Identification of the number of acyl chains involves the formation of heterodimers with TLR6 or TLR1 with TLR2. Heterodimers with TLR1 enables TLR2 to identify triacyl lipoproteins or lipopeptides whilst heterodimerization of TLR2 and 6 is involved in the detection of diacyl lipopeptides (45). However signaling through TLR2 alone has also been reported for some lipopeptides (46).

Inherent properties of lipopeptides such as surfactant, antibacterial and/or antifungal activity have attracted considerable scientific, therapeutic, and biotechnological interests (47). Lipopeptides have been used in the pharmaceutical industry against bacteria and fungi (48, 49). In cosmetics, the surfactant and anti-wrinkle characteristics of lipopeptides are often included in skin care products (50). In the food industry, lipopeptides are used as emulsifiers in various food products (47). Lipopeptides have also been used in biotechnology as biosurfactants, with industrial and medical applications (51–53).

Lipopeptides function to confer a competitive advantage in interactions with other microorganisms by exhibiting lytic and growth-inhibitory activities against a broad range of microorganisms (49, 54), protect against predators (55), allow movement of bacteria across surfaces (56), and enable attachment to surfaces (57). The use of lipopeptides in peptide-based vaccines is derived from the seminal work of Hopp and co-workers in the 1980s which found a significant improvement in antigen-specific antibody response was obtained when a hepatitis virus peptide epitopes was conjugated to a dipalmitoyl-lysine moiety (58). In addition, lipopeptides are well suited to the development of peptide-based vaccines, because they can be easily conjugated to or synthesized during solid-phase peptide synthesis to peptide antigens to generate a highly pure, chemically defined vaccine (59).

Before the discovery of TLRs and their role in lipopeptide recognition, the underlying mechanisms by which lipopeptides elicited an immune response were only partially understood (60). Among several hypotheses, including prevention of enzymatic peptide degradation, it was highlighted that the palmitoyl moiety of lipopeptides may be able to attach and then fuse to APC cell membranes and subsequently deliver peptide epitopes into the APCs (60, 61).

Understanding the mechanism of lipopeptide immunostimulatory activity has motivated research into lipopeptide structure – activity relationships for the development of lipopeptide vaccines. The following section describes the main lipid moieties used and their structure-activity relationships.

## Pam3Cys and Pam2Cys

Braun’s lipoprotein from *Escherichia coli (E. coli)* was identified in the cell-wall of Gram-negative bacteria. The synthetic analog, Tripalmitoyl-*S*-glyceryl-cysteine (Pam3Cys) (Figure 5A), was engineered to enhance the immunogenicity of epitopes derived from influenza virus and enhance virus-specific CTLs when mice were injected with an MHC class I epitope conjugated to Pam3Cys (62–64). Pam3Cys is comprised of three palmitic acid groups that are bound in an ester and amide linkage to a cysteine residue.

This lipid moiety has been studied extensively for the development of peptide vaccines (45, 46, 62, 65, 66). Before applying Pam3Cys as adjuvant, analogs were made and their biological activity tested. Immunological effects of Pam3Cys attached to additional one of five further amino acids were investigated by measuring mitogenicity toward splenocytes and the humoral immune response against the protein conjugate bovine serum albumin (BSA) (67). Out of the analogs (Pam3Cys-Ser-Ser-Asn-Ala, Pam3Cys-Ser-(Lys)_4_, Pam3Cys-Ala-Gly, and Pam3Cys-Ser), Pam3Cys-Ser (Lys)_4_ was the best. Compared to the other analogs, Pam3Cys(Lys)_4_ was more soluble due to the hydrophilic lysine residues, was a potent activator of splenocytes, and optimal for humoral response.

The majority of Pam3Cys studies make use of diastereomeric mixtures. Several studies have revealed that enantiopure Pam3Cys derivatives that contained *R*-configured glycerol were more effective at inducing of cytokine and antibody production in mice when administered with antigens (45, 68, 69). It is likely that the *R*-configuration of the glycerol part of Pam3Cys results in a better interaction with, or has an agonistic effect on, TLR2. The effect of two diastereomers of Pam3Cys-Ser (Lys)_4_ on DCs was investigated in a more recent study (64). The *R*-configured glycerol conjugated to the ovalbumin CTL epitope led to better activation of DCs than the *S*-configured glycerol as determined by higher cytokine secretion and upregulation of DC maturation markers (64). Interestingly, no difference in the uptake of the *R*- and *S*-epimers were observed. Induction of antigen-specific CTL cells was significantly higher in mice administered *S*-epimers than mice that received the *R*-epimers (64). It was concluded that enhanced DC activity was due to improved TLR2 activation by the *S*-epimers of Pam3Cys-Ser (Lys)_4_, resulting in enhancement of the CTL response.

Self-assembled aggregates can function as effective immunogens. Therefore, the stereochemical influence of self-assembly on Pam3Cys and Pam3Cys-Ser were studied. The chirality of the glyceryl moiety and the additional serine unit was found to influence aggregation behavior (65). He more biologically active derivatives contained a serine moiety which formed more thermodynamically stable tubes or rodlike aggregates. This observation was even more pronounced when only one of the two epimers was present.

Synthetic lipopeptide libraries have been used to characterize the contribution of the lipid portion of Pam3Cys to TLR2 specific recognition (66, 67). The activity of Pam3Cys lipopeptides is strongly influenced by the number and type of fatty acids present (66). Cellular responses were abolished with single chain lipopeptide substitution while cytokine activity was significantly reduced for lipopeptides carrying fatty acids with less than 10 carbon chain length (66). Using HEK293 cells expressing recombinant human TLR2, it was demonstrated that the two ester-bound palmitic acids in Pam3Cys mediate a high stimulatory activity while the analog that contained one amide-bound and one ester-bound palmitic acid molecule was inactive (67). In addition, lipopeptide recognition through both murine and human TLR2 depends on the length of the two ester-bound fatty acid chains. These results indicate the importance of the two ester-bound acyl chains rather than the amide-bound acyl chain for TLR2 recognition, as well as a requirement for a minimal chain length (67).

Structure-activity studies with Pam3Cys identified that addition of polar amino acids (e.g., Lys) increased mitogenicity more than Pam3Cys alone and is preferred for use in vaccines. The length of the fatty acid chains plays a marginal role when amide-bound. However ester-bound fatty acid chains have a significant influence on TLR2 activation. Lipopeptides with *R*-configuration in the glyceryl moiety have a higher activity than *S* diastereoisomers.

Toll-like receptor engagement on APC has been demonstrated to influence both primary and secondary TH-cell responses, suggesting that long-term functional capacities of TH cells are determined by innate signals during early stages of infection (70). Induction of influenza hemagglutinin (HA)-specific naive TH cells with HA peptide and the TLR2 agonist Pam3CysK *in vivo* resulted in a high frequency of activated HA-specific TH cells (70). The TLR2-mediated priming also led to an increased frequency of antigen-specific memory TH cells leading to enhanced responses to influenza challenge.

While highly immunogenic and effective at adjuvanting peptide epitopes, lipopeptides that contain Pam3Cys have poor solubility characteristics, making dosing, and formulation difficult. This is predominantly due to the hydrophobic moiety that results from the lipid chains. To overcome this challenge, investigations have focused on the structurally similar derivative, *S*-[2,3-bis(palmitoyloxy)propyl]cysteine (Pam2Cys) that contain one less palmitic acid group and a free amino group with improved solubility characteristics (Figure 5A). Pam2Cys is a synthetic analog of macrophage activating lipopeptide-2 (MALP-2) derived from the cytoplasmic membrane of *Mycoplasma fermentans* (69). Effective immune responses (both antibody and/or cell-mediated) against peptide antigens can also be successfully induced through the use of Pam2Cys (68). Furthermore, Pam2Cys has more favorable solubility properties than Pam3Cys (71). It has also been reported that Pam2Cys is a more potent stimulator of splenocytes and macrophages than Pam3Cys (72–74). The structure-activity relationship between the luteinizing hormone-releasing hormone (LHRH) sequence, a TH epitope, and a lipid moiety (either Pam2Cys or Pam2Cys) was investigated (71). The lipid moiety was attached either to the N-terminus or between the TH epitope and LHRH. The lipopeptide constructs displayed different solubilities and immunological properties that depended not only on the lipid moiety but also on the position of the lipids. Zeng et al. established the most effective vaccine candidate was the most soluble construct which contained Pam2Cys attached at the center of the molecule (71).

Several structural details influence the activity of Pam2Cys as measured by nitric oxide release in macrophages (75). Similar to Pam3Cys, TLR2 was observed to discriminate between the two PAM2Cys stereoisomers; the natural *R* isomer was 100 times more active than the *S* isomer (75). Substitution of the free N-terminus with either an acetyl or palmitoyl group reduces its activity (76). It was concluded that the lack of acyl moiety in mycoplasmal lipopeptides is a key component of their exceptionally high macrophage stimulatory capacity (74). Removal of either (or both) ester-bound fatty acids significantly lowered, or abolished, specific biological activity (76). Substitution of the two ester-bound fatty acids with amide bonds also led to abolition of TLR2 agonist activity (77, 78). The influence of fatty acid length on Pam2Cys activity was also investigated through its ability to activate TLR2 specific cells and induce an antigen-specific humoral response (67, 79). Experimentation in HEK293 cells that expressed recombinant human TLR2 found that the two ester-bound fatty acid lengths should be beyond an alkyl chain of eight carbons (67). Pam2Cys immunogenicity experiments in an *in vivo* BALB/c mouse model found that the LHRH-specific antibody response was dependent on the length of the alkane chains with the order C18 = C16 > C12 > C8 (79). Therefore C16 appears to be the optimal chain length to promote TLR2 recognition of Pam2Cys. In the pursuit of structurally and synthetically simpler Pam2Cys analogs, Agnihotri et al. confirmed the importance of at least one acyl group of optimal length (C16) and the appropriate orientation of the ester carbonyl group. The spacing between one of the palmitoyl ester carbonyl and the thioether was crucial for hydrogen bond formation, which is important in the association between lipopeptides and TLR2 (80).

Pam2Cys based lipopeptides have been studied to compared the quality of cellular immunity generated after lipopeptide vaccination to that observed after influenza A virus infection (81). The number of resident memory CTL in the lungs was equivalent to that observed after viral infection. It was further demonstrated the Pam2Cys moiety was required for efficient expansion of memory CTLs as the CTL recalled with the unlipidated construct was lower (81). This suggests the lipid moiety plays a part in enhancing and maintaining immunological memory. This is further emphasized by human T cells which express high levels of TLR2 after activation and memory T cells express TLR2 and produce cytokines in response to bacterial lipopeptide (82). Therefore, TLR2 and its ligands mediate antigen-specific T cell development and participate in the maintenance of memory T cells.

The lipopeptides illustrated in Figure 5 contain two serine residues between the lipid moiety and the peptide epitopes. It was demonstrated that placement of these serine residues could significantly affect the efficacy of lipopeptides in inducing immune response to peptide epitopes (79). To investigate whether the serine residues acted as spacers or whether they actively contributed to the potency of Pam2Cys lipopeptides they were replaced with another hydrophilic residue (arginine) or with the chemically inert spacer 6-amino hexanoic acid (79). Anti-LHRH antibody titers elicited by the lipopeptides that contained arginine or serine spacers were similar (79). In contrast, anti-LHRH antibody titers induced by lipopeptides containing the more hydrophobic 6-amino hexanoic acid was approximately 10-fold lower. These results suggest that instead of acting solely as spacers, the amino acid situated between lipid and peptide contributed to the adjuvanticity of the lipid moiety with more hydrophilic spacer being optimal (79).

Studies investigating the development of simplified but potent TLR ligands have been aided by the recent publication of co-crystal structures for TLRs and their ligands. Crystal structures of the human TLR1-TLR2-lipopeptide complex and murine TLR2-lipopeptide complex have been published (83). Binding of the tri-acylated Pam3Cys-Ser (Lys)_4_ induces the formation of an “m” shaped heterodimer between TLR1 and TLR2 whereas binding of the di-acylated lipopeptide, Pam2Cys-Ser (Lys)_4_ does not (83). Heterodimerization of the receptors was mediated by the three acyl chains of Pam2Cys-Ser (Lys)_4_ with the two ester-linked lipid chains inside TLR2 while the amide-linked lipid chain resided in a hydrophobic pocket in TLR1 (83). Heterodimer formation was stabilized by hydrogen bonding and hydrophobic interactions. It was hypothesized that formation of the TLR1 and TLR2 heterodimer brings the intracellular TIR domains closer, promoting dimerization and induction of signaling (83).

Structural formulas of various Pam3cys and Pam2Cys based lipopeptide derivatives with the main points of variation in the fatty acids, glycerol moiety, or the cysteine residue have been summarized in detail previously (59). With the focus on more simplified TLR ligands, many groups have utilized simpler acyl moieties for lipopeptide vaccine development. Acyl moieties are more easily synthesized and incorporated into peptide antigens than either Pam2/Pam3Cys. Two of the most studied involve the attachment of palmitic acid and lipoamino acids (Figures 5B,C respectively). The following section will provide information on these most studied simple TLR2 ligands for lipopeptide vaccine development.

## Palmitic Acid

One of the earlier studies used palmitic acid covalently attached to a synthetic peptide from ovalbumin (Ova) to investigate the effect of lipidation on MHC class II-restricted presentation to T cells *in vitro* (84). Palmitoylated Ova was found to activate murine T cells at lower doses than the native antigen (84). It was hypothesized that the palmitoyl group directly participated in stabilizing the peptide displayed on the MHC-TCR complex during antigen presentation (84). These findings had implications for modulating the immunogenicity of synthetic peptides attached to palmitic acid (85).

Investigation of the adjuvanting activity of fatty acid esters highlighted the role of acyl chain length and degree of saturation on the humoral response to BSA and staphylococcal toxoid in mice (86). Adjuvant activity was increased by changing the chemical properties of the esters by: (i) using ethyl esters where the acyl chain length of the fatty acid component was 16 or greater and isobutyl and isopropyl esters of palmitic acid (C16: 0) were superior to ethyl esters (86).

Palmitoylated peptides have been assessed as vaccine candidates for numerous diseases, some of which have been evaluated in clinical trials (87). Development on an anti-human immunodeficiency virus (HIV) lipopeptide vaccine used peptides derived from regulatory or structural HIV-1 proteins (Nef, Gag, and Env) modified by C-terminal addition of a single palmitoyl moiety (87, 88). A phase I study to evaluate immunogenicity and tolerance of the lipopeptide vaccine in human volunteers given three injections of the lipopeptide found that the vaccine elicited strong multiepitopic B and T cell responses (88). Production of immunoglobulin G (IgG) antibodies that recognized the Nef and Gag proteins was also observed (88).

Mapping of B and T cell epitopes has allowed the lipopeptide vaccine approach to be extended to the most relevant epitopes within the pre-erythrocytic stage of malaria (89). In a pre-clinical study in chimpanzees (genetically the closest primate relative of humans), the feasibility of a palmitoylated peptide derived from malarial liver-stage antigen-3 targeting infected hepatocytes has been demonstrated (90). This approach used mixtures of synthetic antigens from liver-stage antigen-3 to induce protective immunity (90).

A lipopeptide-based vaccine for human cytomegalovirus (HCMV) that involves the lipidation of a HCMV CTL epitope derived from the immunodominant pp65 protein linked to a universal TH epitope, induced antigen-specific CTL response (91). These CTLs recognized, and were capable of lysing, HCMV-infected human cells.

Induction of CTL mediated long-term influenza virus clearing responses by lipopeptides had been examined (92). The most potent immunogen for eliciting pulmonary viral clearing responses contained peptides representing determinants for TH and CTL peptides epitopes together with two or four palmitoyl groups. The lipopeptides and the non-lipidated analogs induced equivalent levels of cytolytic activity in the primary effector phase of the response when administered with additional adjuvant. The ability to recall lytic responses, however, diminished much more rapidly in non-lipidated analogs. By 15 months postpriming, lytic activity in lipopeptide-inoculated mice remained potent unlike non-lipidated peptide-primed mice. The HBV-specific CTL responses induced in humans by lipopeptide vaccination were found to be a safe and effective means of generating significant memory CTL responses (93). CTL responses induced were similar magnitude to patients who successfully cleared HBV infection induced by natural exposure. *Plasmodium falciparum* liver-stage antigen with the addition of a palmitoyl chain were shown to increase immunogenicity and lead to long lasting antibody production up to 8 months (94). B and T cell responses induced by this lipopeptide were reactive with native parasite protein epitopes and was safe and highly immunogenic in chimpanzees (94), a primate with an immune system similar to that of humans. The ability to induce CTL responses including the magnitude, consistency, and memory of CTL responses in chimpanzees demonstrated significant CTL responses 9 months after the final immunization (89). The percent specific lysis had only decreased from 22.3 to 18.7% for the best lipopeptide vaccine candidates (89). The murine influenza virus CTL epitope NP 147–155 as a model system covalently attached to two palmitic acids was found to be highly immunogenic, and a single injection resulted in memory CTL induction that persisted for more than 1 year (95). These studies demonstrate lipopeptides safely induce specific memory immune response in mice and humans of such magnitude and persistence as to be of therapeutic importance.

Mucosal administration of lipopeptide vaccines rather than standard parenteral injections is advantageous from a safety, logistical, and cost point of view (96). Furthermore, non-invasive mucosal immunization allows the possibility to induce mucosal immunity concurrently with systemic immunity, while systemic immunization does not induce mucosal immunity. Lipidation is essential in directing mucosally administered antigens across the mucosal epithelial layer (96). The mucosal surface is the first line of defense against many pathogens and immunity at this local site could prevent infection before the disease develops. A herpes simplex virus type 2 (HSV-2) CTL epitope conjugated to a palmitic acid moiety was delivered mucosally and generated HSV-2-specific CTLs both locally in the genital tract and at systemic sites (97). Thus lipopeptides as novel mucosal vaccines have attractive practical and immunological benefits (98).

It has been established that synthetic mimics of bacterial lipopeptides (Pam3/Pam2Cys) exert their self-adjuvanting activity through TLR2, less is known about how lipopeptide vaccine candidates that contain palmitic acids exert their immunological effect. These lipid components bear little structural similarity to bacterial lipopeptides apart from the presence of an acyl moiety. However, it has been shown that lipopeptides that contain a single palmitic acid enhance uptake, maturation, and production of pro-inflammatory cytokines release by DCs in a TLR2 specific manner (97, 99). This highlights how simpler lipid structures may be employed for the development of TLR2-targeting peptide vaccines.

## Lipoamino Acid

An antibody response against peptide epitopes can be elicited by synthesis of the epitope from lipoamino acids (LAA) (Figure 5C). Lipoamino acids are alpha-amino acids with long hydrocarbon side-chains that combine the structural features of lipids with those of amino acids (100). Their ease of synthesis and the ability to modify the hydrocarbon side-chain (lipid character) makes LAAs ideal for enhancing peptide immunogenicity.

A subsequent application of this technology has been the development of the lipid-core peptide (LCP) system (Figure 5C), which incorporated the lipoamino acid-based adjuvant into a polylysine system to enhance the immunogenicity of peptides (101). Incorporation of two or three copies of LAAs, with glycine spacers allows mimicry of the Pam2Cys and Pam3Cys structure.

A preliminary study with LAAs as adjuvants showed that attachment with peptide-epitope significantly enhanced immunogenicity (up to 3200-fold) (102). LAA were attached to epitopes derived from the variable domains (VD) of *Chlamydia trachomatis* (*C. trachomatis*) outer membrane protein. A high titer antibody response was generated against three serovars of *C. trachomatis* that cause trachoma (102).

The LAA-based platform has been used to immunize against group A streptococci (GAS) (101, 103, 104), the causative agent of rheumatic fever and rheumatic heart disease. In an extensive structure-activity study to enhance the immunogenicity and optimize the constructs to stimulate immunity against GAS M protein specific p145 peptide epitopes, a library of different LCP constructs were synthesized and the immunogenicity of each compound examined (105). The most immunogenic constructs contained the longest alkyl side-chains for the LAAs (18 carbons). The number of copies of LAAs in the constructs affected the immunogenicity and spacing between the LAAs increased immunogenicity. Some constructs without external adjuvant were more immunogenic than the p145 peptide administered with complete Freund’s adjuvant (CFA) (105). CFA is the gold standard for adjuvant efficacy but restricted to animal use due to its toxicity. These data showed the potential for LCP based lipopeptides to enhance immunogenicity of GAS derived peptides.

Development of self-adjuvanting lipopeptide vaccine candidates composed of three components: a GAS B cell epitope (J14), a TH epitope derived from canine distemper virus, and an immuno-stimulant based on LAA showed that the orientation of the three components affected the J14-specific immune response (antibody titer) (106). Correlations between spatial arrangements of the three components and both the vaccine secondary structure (conformation) and level of protection against GAS infection was identified in a later study (107).

LAA-based lipopeptide vaccine candidates have been designed, synthesized, and immunologically evaluated for a number of other diseases. Constructs incorporating a cluster of the most common tumor-associated carbohydrate antigens (known as Tn antigen) covalently attached to T cell peptide epitopes and LAA consisting of two 16-carbon lipoamino acids have been evaluated (108). These constructs induced potent antibody responses in mice without the need for an additional adjuvant, carrier protein, or special formulation (e.g., liposomes). Structure-activity studies demonstrated that linear or branched vaccine architecture had a significant effect on antibody recognition (108).

In an attempt to develop a vaccine to target the most prevalent human hookworm, *Necator americanus*, a B cell peptide-epitope from the apical enzyme in the hemoglobin digestion cascade (the aspartic protease Na-APR-1) was incorporated into the LCP system. The LCP construct induced a strong IgG response in mice and antibodies produced were able to bind to and completely inhibit the enzymatic activity of Na-APR-1 *in vitro*. The results presented show that the construct can induce enzyme-neutralizing antibodies in mice.

Evaluation of whether LAA-based lipopeptides that incorporate TH and CTL epitopes could induce epitope-specific T cell responses and protect against pathogen challenge in a rodent malaria model has recently been undertaken (109). The vaccine constructs failed to induce an expansion of antigen-specific response. However CTL responses of unknown specificity were induced which were able to protect against parasite challenge (109). These data demonstrate that vaccination with LAA-based vaccine candidates can confer non-specific protective immunity against *Plasmodium* parasite challenge.

LAA-based lipopeptides have been used extensively for the development of a mucosally active GAS vaccine (110, 111). Immunological evaluation in mice demonstrated that the point of epitope attachment and the length of the LAA alkyl chain have a profound effect on vaccine immunogenicity after intranasal administration (111). It was demonstrated that a vaccine featuring a C-terminal lipid moiety that contained alkyl chains of 16 carbons; a TH epitope located at the N-terminus; and J14 attached to the side-chain of a central lysine residue, was capable of inducing optimal antibody response and mucosal IgA response (110, 111). Through structure-activity studies, a GAS vaccine candidate that induced J14-specific mucosal and systemic antibody responses when administered intranasally without additional adjuvants was identified. The systemic antibodies elicited inhibited growth of GAS *in vitro* (111). J14-specific mucosal antibody titers corresponded with reduced throat colonization after GAS respiratory challenge (111).

The self-adjuvanting effect of LAA-based lipopeptides was demonstrated to act through stimulation of TLR2 and DC activation (110, 112). Investigation to determine whether LAA-based lipopeptides activated NF-κB in a TLR2 dependent manner found that activation was dose-dependent and affected by the length of the alkyl chains of the incorporated lipid moieties with the hierarchy 16 carbons > 14 carbons > 12 carbons (113). Two copies of LAA was more effective for NF-κB activation that one or three copies (113). To determine whether lipopeptides were recognized by DCs *in vivo*, cohorts of mice were administered LCP constructs. Splenic DCs were isolated and maturation assessed by measuring MHC class II expression. It was found that the expression of MHC class II increased by day 3, and that co-stimulatory molecules were upregulated (114). It was also determined that LCP was capable of signaling through human TLR2 in an *in vitro* based assay (114). LAA-based lipid moieties have significant adjuvant activity and are able to induce the maturation of murine DCs, potentially by signaling via TLR2.

## Conclusion

Peptide-based vaccines offer several advantages over the conventional vaccines in terms of purity and specificity of the immune response elicited. However, the use of often toxic adjuvants, which are critical for immunogenicity of synthetic peptides, have hampered their progress. Improvements in immunogenicity through lipidation of peptides discovered several decades ago is a promising approach. Several pre-clinical and clinical trials have revealed that lipopeptide vaccines are effective, safe, and can be synthesized based on minimal peptide epitopes using peptide synthesis methods to combat wide variety of infectious diseases.

More recent scientific advances have identified mechanism that lipopeptide self-adjuvanting activity occurs through the PRR TLR2. Mammalian TLRs are expressed on macrophages and DCs which are primarily involved in mediating immune responses. TLRs are critical for sensing invading pathogens and recognition by TLRs provokes rapid activation of innate immunity through induction of inflammatory cytokines and upregulation of co-stimulatory molecules. This subsequently leads to effective immunity. In this regard, TLRs are adjuvant receptors and have allowed the development of promising lipopeptide vaccine candidates. Rational vaccine design, including optimization of immune response through structure-activity studies, allows the elicitation of predictable immune responses to combat pathogens. In particular, the recent mucosal application of lipopeptide vaccines represents an ideal strategy against many pathogens as the mucosal surfaces represents a primary infection site. Using non-invasive mucosal routes would be highly advantageous for vaccination programs, since mucosal administration is simple, needle-free, and cheap.

With our recent understanding of how the lipid component confers the “self-adjuvanting” activity of lipopeptides and the development of simple lipid moieties, lipopeptide could form the basis for vaccine development against numerous diseases.

## Conflict of Interest Statement

The authors declare that the research was conducted in the absence of any commercial or financial relationships that could be construed as a potential conflict of interest.
